# *Lactobacillus rhamnosus* AD3 as a Promising Alternative for Probiotic Products

**DOI:** 10.3390/biom11010094

**Published:** 2021-01-13

**Authors:** Aldo Stivala, Giuseppe Carota, Virginia Fuochi, Pio Maria Furneri

**Affiliations:** Department of Biomedical and Biotechnological Sciences (BIOMETEC), University of Catania, 95123 Catania, Italy; astivala@unict.it (A.S.); giuseppe-carota@outlook.it (G.C.); furneri@unict.it (P.M.F.)

**Keywords:** *L. rhamnosus*, antimicrobial activity, probiotic properties, urogenital infections, *S. agalactiae*, *Candida* spp.

## Abstract

*Lactobacillus* strains dominate the vaginal habitat and they are associated with a lower risk of genital infections. In addition, they contribute to the conservation of the vaginal microbiota by producing postbiotic agents. Previous studies have shown that their predominance involves antimicrobial activity against urogenital pathologies. In this context, probiotics may improve treatment outcomes. The aim of this study was to evaluate the probiotic properties of lactobacilli strains of vaginal origin using a multidisciplinary approach. For this purpose, safety criteria, ability to resist at low pH and bile salts, antimicrobial activity, ability to produce biofilm, capacity to produce hydrogen peroxide and more importantly, auto-aggregation, co-aggregation (with *Candida* spp.) and adhesion to human cells were evaluated. The strains belonged to the species of *L. crispatus*, *L. gasseri*, *L. rhamnosus* and *L. delbruckii.* Among these, a strain of *L. rhamnosus* named AD3 showed the best probiotic properties. As probiotics are already in use in many clinical practice and there are no major safety concerns, *L. rhamnosus* AD3 showed promise in becoming a prevention and complementary treatment option for urogenital diseases. Indeed, these results suggest that strain *L. rhamnosus* AD3 is non-pathogenic and likely to be safe for human consumption. This study revealed the great amensalistic properties of a new *L. rhamnosus* strain which can aim to be used as probiotic in pharmaceutical applications.

## 1. Introduction

Urogenital infections are common concerns in young women [1,2]. Among all sexually transmitted diseases, bacterial vaginosis and vulvovaginal candidiasis are the most frequent [3,4]. These infections make-up the most common reasons for a woman to need to visit to gynaecologist, with the outcome of discomfort and also enormous costs for health care treatment [5]. Moreover, these kinds of diseases most often include treatment with antibiotics and/or antimycotics, which have led to a raised concern regarding drug resistance among pathogenic species [6,7].

The human vaginal ecosystem is dominated by *Lactobacillus* species and an altered vaginal microbiota can cause a greater chance of symptomatic conditions. Furthermore, there is a clear relationship between vaginal microbiota and urogenital infections [8], in fact most of the microorganisms in the vagina also derive from the gastrointestinal tract, and this does not depend only on sexual activity but also on personal hygiene and nutrition [9]. The protective role of lactobacilli is increasingly accepted and several clinical studies are underway to evaluate the use of lactobacilli as probiotics. Further studies have shown that the composition of the vaginal microbiota can undergo changes throughout life. The vaginal environment is dynamic and it changes in relation to events such as sexual activity, the menstrual cycle or menopause [10]. During the fertile age, there is a vaginal epithelium rich in glycogen and mucins that act as nutrients and that allow colonization and domination of the lactobacilli. Furthermore, the fertile age is characterized by monthly ovulation and therefore high estrogenic levels which, also thanks to the action of the proton pumps, bring the vaginal lumen to be particularly acidic, which leads to better conditions for the growth of the lactobacilli [11]. In postmenopausal women, especially in those who show signs of vaginal dryness and atrophy, a reduction in the vaginal Lactobacilli component can be noted [12]. Sexual activity can also influence the vaginal microbial composition, causing a reduction in the lactobacilli component with relative loss of their protective action, and an increase in the predisposition to dysbiosis [13]. 

In this context, the use of probiotics consisting of *Lactobacillus* spp. is increasing in the gynaecological field to restore the physiologic vaginal microbiota and treat bacterial vaginosis and vulvovaginal candidiasis even if there is conflicting evidence between them [14,15,16]. Although the local administration should be the best and fastest solution to the problem of vaginal dysbiosis, main scientific concept associated with the use of probiotics is the oral administration of live microorganisms in order to restore the environment by competition [17]. 

Of the many probiotic microorganisms available commercially, *L. rhamnosus* GG and *L. rhamnosus* GR-1 are the most studied and they have been used successfully to treat and prevent diarrhoea and urogenital infections in women, respectively [18,19].

Nonetheless, the strains of Lactobacilli used in such treatments must respect certain rules so that they can be considered potential probiotics: they should be able to adhere to the cells, to prevent or decrease adherence of pathogenic microorganisms, to secrete acids, hydrogen peroxide, and bacteriocins so as to prevent growth of pathogens. Moreover, they should be safe, and should not be invasive, carcinogenic, and pathogenic and finally, they should be able to form clusters so as to produce normal-balanced microbiota [20]. 

In view of the fact that the market can offer different products with various probiotic components, the main goal of the study had been the characterization of *Lactobacillus* strains in order to clarify their ecological role in the female genital tract and to focus on selected *Lactobacillus* strains with probiotic properties to finding a valid prevention and complementary treatment option for urogenital diseases, and to improve the normal vaginal microbiota in healthy women. Moreover, the project included in vitro studies on *L. rhamnosus* GG for a comparison on the probiotic properties of the strains investigated.

## 2. Materials and Methods 

### 2.1. Bacterial Strains and Growth Conditions

In this study, twelve strains of *Lactobacillus* spp., previously isolated from vaginal swabs, were used. *L. rhamnosus* GG was used as internal positive control for probiotic properties. 

Moreover, ATCC strains of pathogenic bacteria were used to assess antibacterial activity: *E. coli* ATCC 25922, *E. faecalis* ATCC 29212, *P. aeruginosa* ATCC 27853, *S. agalactiae* ATCC 13813 and *K. pneumoniae* ATCC 700603. Finally, clinical strains of *Candida* spp., previously isolated from women with vulvovaginal candidiasis, were used to assess antifungal activity: *C. albicans*, *C. glabrata* and *C. tropicalis*. All strains are available to Laboratory of Applied Microbiology, Department of Biomedical and Biotechnological Sciences, Università degli Studi di Catania.

*Lactobacillus* spp. were grown in de Man, Rogosa and Sharpe (MRS) broth (Oxoid, Thermo Fisher Scientific Inc., Rodano (MI), Italy, CM0359) at 37 °C for 48 h under micro-aerobic conditions before proceeding with subsequent investigations. *Candida* strains were grown on Sabouraud Dextrose Agar with Chloramphenicol (Oxoid, Thermo Fisher Scientific Inc., Rodano (MI), Italy, PO0358) at 35 °C for 48 h under aerobic conditions before proceeding with subsequent investigations and finally, bacteria strains were grown in Muller-Hinton (MH) broth (Oxoid, Thermo Fisher Scientific Inc., Rodano (MI), Italy, CM0405) at 37 °C overnight before proceeding with subsequent investigations.

### 2.2. LAB Identification

#### 2.2.1. DNA Extraction

RTP Bacterial DNA Mini Kit (Stratec Biomedical AG, Birkenfeld, Germany) was used to extract bacterial DNA following manufacturer instruction. Then, DNA samples obtained were visualized by electrophoresis (70 V) in agarose gel (1.5%).

#### 2.2.2. Molecular Identification

16S rDNA PCR/RFLP and, multiplex-PCRs to amplify different loci of the 16S-23S gene region and ITSregion flanking the 23S rDNA gene were used to make molecular identification as previously described [21].

### 2.3. Safety Criteria of Potential Probiotics

#### 2.3.1. Antibiotic Resistance

Ampicillin, erythromycin, tetracycline, gentamicin, clindamycin, chloramphenicol, streptomycin and kanamycin, were used to assess antibiotic resistance according to European Food Safety Authority [22]. Moreover, ciprofloxacin was used as internal negative control. 

#### 2.3.2. Gelatinase Activity and Haemolytic Activity

Gelatinase activity and haemolytic activity were evaluated as previously described [23]. Briefly, overnight cultures of strains were streaked onto Nutrient Gelatin agar plates (Oxoid, Thermo Fisher Scientific Inc., Rodano (MI), Italy, CM0635) and Columbia +5% sheep blood agar plates (bioMérieux Corporate, Bagno a Ripoli (FI), Italy). Plates were incubated at 37 °C under micro-aerobic conditions for 48 h. *Bacillus subtilis* ATTC 23857 and *Streptococcus pyogenes* ATCC 19615 were used as internal positive control for gelatinase activity and haemolytic activity, respectively.

### 2.4. Evaluation of Probiotic Properties

#### 2.4.1. Ability to Resist at Low pH and Bile Salts

The evaluation of the resistance to low pH and bile salts was carried out by bacterial counts and relative survival rate after stressed growth [24]. Briefly, *Lactobacillus* strains and *E. coli* ATCC 25922 (as positive internal control) were grown in LSM broth and MH broth, respectively, at 37 °C overnight. Then, two different conditions were evaluated: growth in bile salts agar, and growth in bile salts agar after one hour in acidic condition (pH 3.0). Subsequently, serial ten-fold dilutions in NaCl (0.85% *w*/*v*) were made and plated on agar for enumeration. Each experiment was made six times in duplicate and the growth in LSM broth (and MH broth for *E. coli*) without stress was used as control. 

#### 2.4.2. Antimicrobial Activity

Cells-free supernatants produced by *Lactobacillus* strains (grown at 37 °C, 18 h in micro-aerobic condition) were neutralized with NaOH 1 mol·L^−1^ and inoculated into wells in pre-inoculated MH agar plates and Sabouraud dextrose agar with chloramphenicol, with bacteria and fungi, respectively [25,26,27]. For quality control ciprofloxacin and itraconazole were used as positive internal controls for antibacterial and antifungal activity, respectively. MH plates were incubated at 37 °C overnight in aerobic condition to evaluate antibacterial activity, and SAB plates were incubated at 33 °C for 24 h in aerobic condition to evaluate antifungal activity. Inhibition zones were measured by a gauge [23,28]. Results were expressed in mm ± SD.

#### 2.4.3. Ability to Produce Biofilm 

*Lactobacillus* strains were tested for the ability to form biofilm. An inoculum (1.5 × 10^5^ CFU mL^−1^) of each strain was incubated in MRS broth at 37 °C under micro-aerobic condition. Then, the absorbance at 600 nm (BioRad Microplate Reader Model 680, ©2021 Bio-Rad Laboratories, Inc., Segrate (MI), Italy) was recorded and the broth was discharged. The plates were washed, with PBS in order to remove suspended cells, and incubated at 60 °C for 1 h. Subsequently, 200 µL of crystal violet were added in each well and after 10/15 min crystal violet residues were dissolved with a solution of methanol/acetic acid 2:1.5. A second read was recorded at 570 nm. The following formula

OD_570_/OD_600_ × 0.4

was used to calculate biofilm and OD 0.061 was considered as cut-off value.

#### 2.4.4. Capacity to Produce Hydrogen Peroxide

Horseradish peroxidase (Sigma, Merck KGaA, Darmstadt, Germany, P6782) and 3,3′,5,5′-tetramethylbenzidine (Sigma, Merck KGaA, Darmstadt, Germany, T2885) were added to MRS agar to evaluate and quantify hydrogen peroxide production by *Lactobacillus* strains. Briefly, a bacterial inoculum (1.5 × 10^8^ CFU mL^−1^) was streaked on this MRS modified agar and, the plates were incubated under micro-aerobic condition for 48 h at 3 °C, and further at room temperature for other 24 h in the dark. Negative producers gave colourless colonies, while positive producers gave colour colonies from brown to dark blue.

#### 2.4.5. Ability to Adhere to Human Hep-2 and Caco-2 Cell Lines

Hep-2 cells (Human epithelial type 2 cells, human laryngeal carcinoma, ATCC® CCL-23™, University Boulevard Manassas, VA, USA) and Caco-2 cells (Human epithelial cells, human colorectal adenocarcinoma, ATCC® HTB-37™, University Boulevard Manassas, VA, USA) were cultured in DMEM (Dulbecco’s MEM—Biochrom GmbH, Berlin, Germany). Medium were supplemented with 6% FBS, 2 mM L-glutamine, 100 U·mL^−1^ of penicillin and 100 μg·mL^−1^ of streptomycin. Plates were incubated at 37 °C in a humidified, 95% air/5% CO_2_ atmosphere. The medium was changed every 2–3 days.

One day before the experiment, cells were trypsinized, counted and inoculated in 129AX-1 tubes with a slide (Sterilin^TM^, Thermo Fisher Scientific Inc., Rodano (MI), Italy); approximately 1.5 × 10^5^ cells/tube. After 48 h the cells were washed in PBS, and a bacterial suspension (1.0 × 10^8^ CFU mL^−1^) was added and the mixture incubated for 30 min at room temperature. Then, three washes with PBS were done and cells were fixed with ethanol 1 mL/tube for 15 min at 3 °C, stained with 20% Giemsa (*v*/*v* in phosphate-buffered water) for 20 min (in the dark) and examined microscopically under oil immersion. For each slide 200 cells were observed and the results are expressed as the average of bacteria per cells. The adhesion test was repeated at least six times and data were summarized using mean (±SD/cell) [29].

#### 2.4.6. Auto-Aggregation Study

Auto-aggregation study was performed with slightly modified protocol previously described by Gil et al. [30]. Briefly, the strains were grown overnight at 37 °C in MRS agar, then suspensions of 0.6 at 600 nm (BioRad Microplate Reader Model 680, © 2021 Bio-Rad Laboratories, Inc., Segrate (MI), Italy) in PBS were done. Variation of OD was monitored every hour for 4 h. Then, the percentage of auto-aggregation was calculated as follows:

% Autoaggregation = (OD*_in_* − OD*_fin_*/OD*_fin_*) × 100

Gram staining was used to visualize the aggregates under oil immersion microscopy (1000×).

#### 2.4.7. Co-Aggregation Study

Co-aggregation study was evaluated as follows: 129AX-1 tubes with slides (Sterilin^TM^, Thermo Fisher Scientific Inc., Rodano (MI), Italy) were added with 500 µL of an overnight culture of *Lactobacillus* strains grown at 37 °C in MRS broth (Oxoid, Thermo Fisher Scientific Inc., Rodano (MI), Italy, CM0359) under micro-aerobic conditions and 500 µL of an overnight culture of *Candida* spp. grown at 33 °C in DMEM (Dulbecco’s MEM—Biochrom GmbH, Berlin, Germany). Tubes were incubated at 37 °C for 4 h. Then, Gram staining was used to visualize the co-aggregates under oil immersion microscopy (1000×) [30]. Scoring was done according to Reid et al. [31]. Moreover, the co-aggregation were also evaluated on Hep-2 and Caco-2 cells. Tubes with monolayer cells were incubated at 37 °C for 4 h after inoculation with *Candida* and *Lactobacillus* strains, then Giemsa staining was used to visualize co-aggregates and adhesion on cells. 

### 2.5. Statistical Analysis

All experiments were performed at least three times and data were summarized using mean (±SD). Where applicable, data were analysed by one-way ANOVA with correction for multiple comparisons by Bonferroni. All results with *p*-value < 0.05 were considered significant. All results and graphs were generated using GraphPad^®^ Prism ver. 6 software.

## 3. Results

### 3.1. LAB Identification

The identification of the species of *Lactobacillus* isolates was conducted with the amplification of 16S rDNA. Four species were identified among which *L. crispatus* was the most representative (6 strains), followed by *L. gasseri* (4 strains), *L. rhamnosus* (1 strain) and *L. delbrueckii* (1 strain). 

### 3.2. Safety Criteria of Potential Probiotics

#### 3.2.1. Antibiotic Resistance

Antibiotic resistance was evaluated to verify if the twelve *Lactobacillus* strains could be accepted to use as potential probiotic. Strains were inhibited by the eight antibiotics tested according to the microbiological cut-off values defined by EFSA (Table 1). Therefore, all strains were susceptible to all antibiotics. Concurrently, the strains resulted resistant for the antibiotic CIP used as quality control.

#### 3.2.2. Gelatinase Activity and Haemolytic Activity

Gelatinase activity was not found for the *Lactobacillus* strains tested. Furthermore, lysis on blood agar plates were not shown for any *Lactobacillus* strain under examination. 

### 3.3. Evaluation of Probiotic Properties

#### 3.3.1. Ability to Resist at Low and Bile Salts

**Figure 1:**
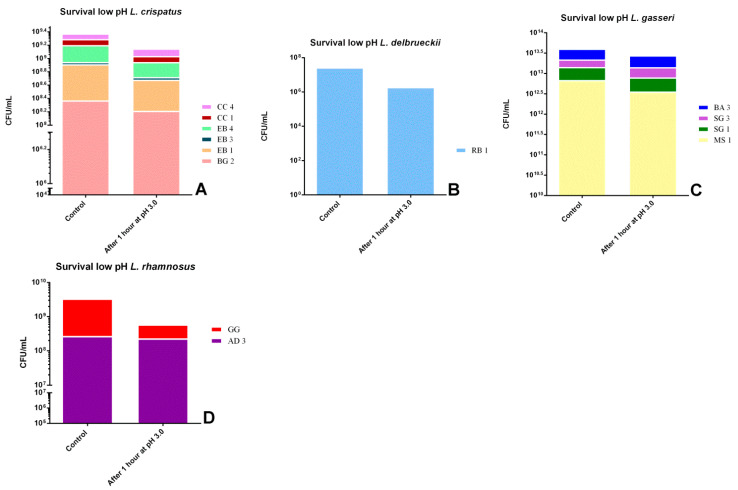
Comparison between control growth and growth after one hour of exposure to pH 3 by associating the strains by species: (**A**) *L. crispatus* strains,(**B**) *L. delbrueckii* strain, (**C**) *L. gasseri* strains and (**D**) *L. rhamnosus* strains.

**Figure 2:**
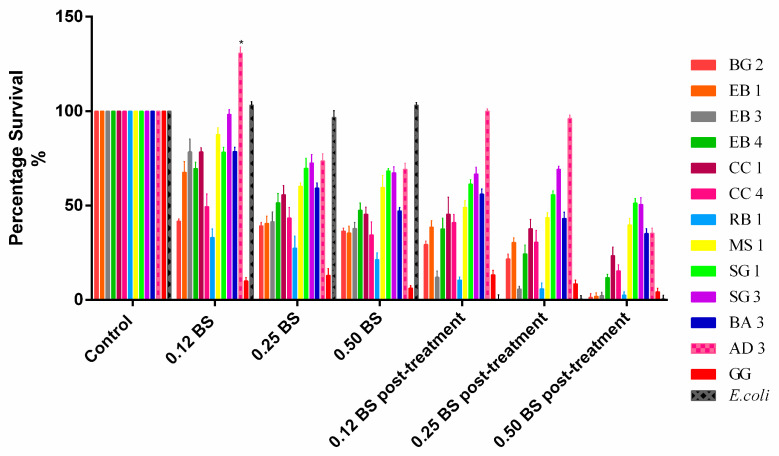
Percentage of survival at different concentrations of bile salts before and after exposure to acid pH. Data are showed as mean ± SD; *p* values were calculated by applying one-way ANOVA with Bonferroni correction for multiple comparisons. Significance vs. control * *p* < 0.05.

As already stated, according to FAO [32], bacterial strains must resist to gastrointestinal conditions and remain viable to be considered probiotics. Figure 1 and Figure 2 show the reduction to exposition to low pH and different concentrations of bile salts, respectively. First of all, all strains survived one hour at pH 3.0 but with substantial differences: the strain that showed the greatest resistance was *L. rhamnosus* AD3 (−15.39%), followed by *L. gasseri* SG3 (−18.16%) and *L. crispatus* CC4 (−21.95%). In contrast, the most susceptible strains were *L. rhamnosus* GG (−88.33%) and *L. delbrueckii* RB1 (−99.28%). 

All isolates showed good tolerance to bile salts at difference concentration pre-exposition at low pH. In particular, *L. rhamnosus* AD3 proved to be the most resistant, with a survival of 69.2% with bile salts at 0.5% and even 130.8% with bile salts at 0.12%. Oddly, the only strain that showed significant reduction in bile salt treatment was *L. rhamnosus* GG (−93.7% with BS 0.5%; −90% with BS 0.12%). After exposure to acid pH for one hour, the consequent exposure to bile salts showed a slight decrease for all strains including *L. rhamnosus* GG. Additionally in this case, *L. rhamnosus* AD3 proved to be the most resistant strain (−0%). This means that after GI tract passage, all strains were able to survive but not at the same initial concentration of inoculum. 

#### 3.3.2. Antimicrobial Activity

The antagonistic activity of putative probiotic strains was assessed against the following target microorganisms: *Escherichia coli* ATCC 25922, *Enterococcus faecalis* ATCC 29212, *Pseudomonas aeruginosa* ATCC 27853, *Klebsiella pneumoniae* ATCC 700603, a clinical isolate of *Streptococcus agalactiae*, three clinical isolates of *Candida* (*C. albicans* 10, *C. glabrata* 14, *C. tropicalis* 21). The strains showed good activity both against Gram-positive and Gram-negative pathogens, and also excellent activity against *Candida* strains (Table 2). *L. gasseri* SG3 showed the highest antagonistic activity against both bacteria and fungi, followed by *L. rhamnosus* AD3 which however had no effect against *E. coli* and *C. tropicalis.* Finally, no inhibition zones were shown by *L. rhamnosus* GG.

#### 3.3.3. Ability to Produce Biofilm

**Figure 3:**
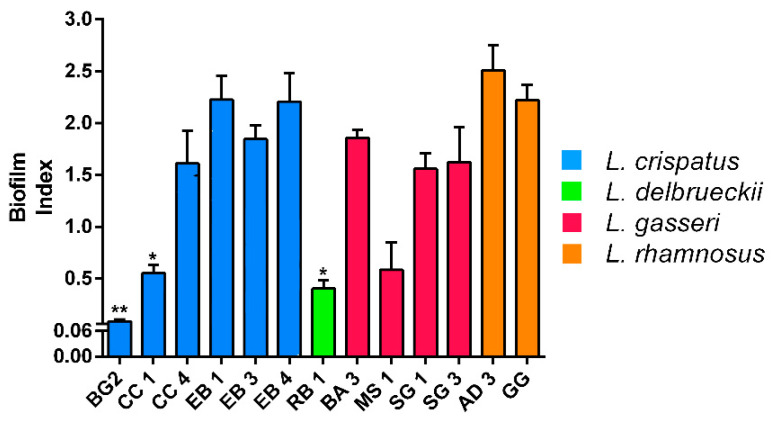
Biofilm formation by *Lactobacillus* strains recorded after overnight growth at 37 °C under microaerobic condition. Data are showed as mean ± SD; *p* values were calculated by applying one-way ANOVA with Bonferroni correction for multiple comparisons. Significance vs. AD 3 and GG * *p* < 0.05, ** *p* < 0.01.

The strains were divided by means of BI: non-producers (OD < 0.061), weak producers (0.061 < OD < 0.120), medium-sized producers (0.121 < OD < 0.300) and strong producers (OD > 0.300) [23]. All strains showed moderate biofilm production after overnight growth at 37 °C, with the exception of *L. crispatus* BG2 which instead proved to be the weakest producer (Figure 3).

#### 3.3.4. Capacity to Produce Hydrogen Peroxide

**Figure 4:**
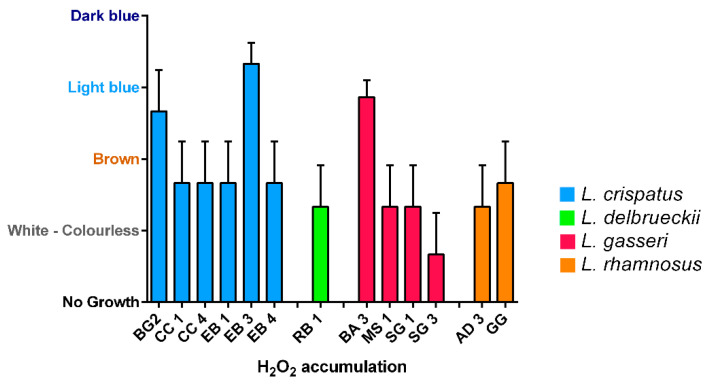
Hydrogen peroxide production of *Lactobacillus* isolates. White-colorless: negative; Brown: slightly positive; Light blue: moderate; Dark blue: strong. Data are showed as mean ± SD.

Production of hydrogen peroxide was tested in 12 vaginal lactobacilli strains and *L. rhamnosus* GG by the semi-qualitative TMB peroxidase assay (Figure 4). Only three strains out of 13 produced great amount of hydrogen peroxide: two *L. crispatus* (BG2, EB3) and one *L. gasseri* (BA3). *L. rhamnosus* GG showed a moderate production. 

#### 3.3.5. Ability to Adhere to Human Hep-2 and Caco-2 Cell Lines

The ability of a bacterial strain to adhere to human epithelial cells is one of the most important properties for such a strain to be considered probiotic. Indeed, this feature will allow it to colonize the mucous membrane and perform important modulating functions on the immune system. In this study, *Lactobacillus* strains were tested for adhesion to Hep-2 and Caco-2 cells (Table 3). Among all isolates tested, *L. rhamnosus* AD3 and *L. crispatus* EB1 showed heavy in vitro adhesion towards both epithelial cell lines compared to *L. rhamnosus* GG (≈47/cell). 

#### 3.3.6. Auto-Aggregation Study

**Figure 5:**
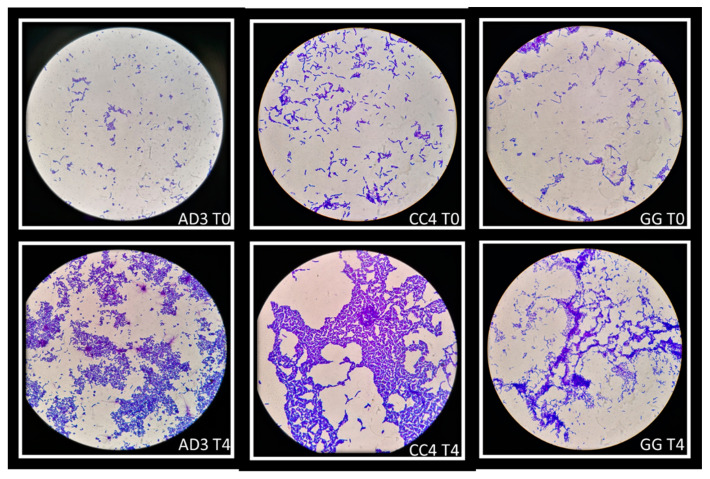
Auto-aggregation of vaginal lactobacilli at two time-points: T0 and T4. *L. rhamnosus* AD3, *L. crispatus* CC4 and *L. rhamnosus* GG showed greater ability to self-aggregate than others strains. Strains were observed under the microscope (1000×) after Gram staining.

The ability of auto-aggregation of vaginal lactobacilli was evaluated at five time-points (0–4 h). This ability was confirmed at all time-points but the strains showed the highest degree of auto-aggregation after 4 h of incubation. Some strains have a greater ability to self-aggregate than others: in this study *L. rhamnosus* AD3 and *L. crispatus* CC4 showed the highest auto-aggregating aptitude, even greater than *L. rhamnosus* GG. Figure 5 showed auto-aggregation observed for *L. rhamnosus* AD3, *L. crispatus* CC4 and *L. rhamnosus* GG in two time-points (T0 and T4). 

#### 3.3.7. Co-Aggregation Study 

**Figure 6:**
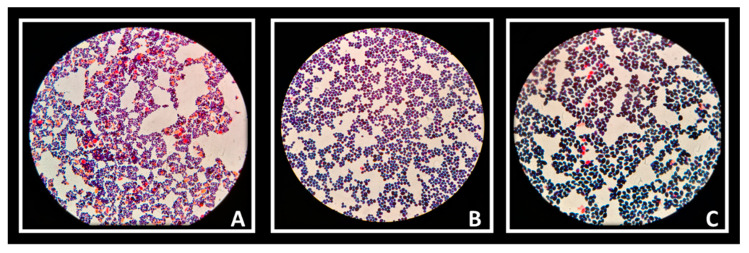
*Candida albicans* (**A**), *C. krusei* (**B**) and *C. tropicalis* (**C**) observed under a microscope (1000×) after Giemsa staining.

**Figure 7:**
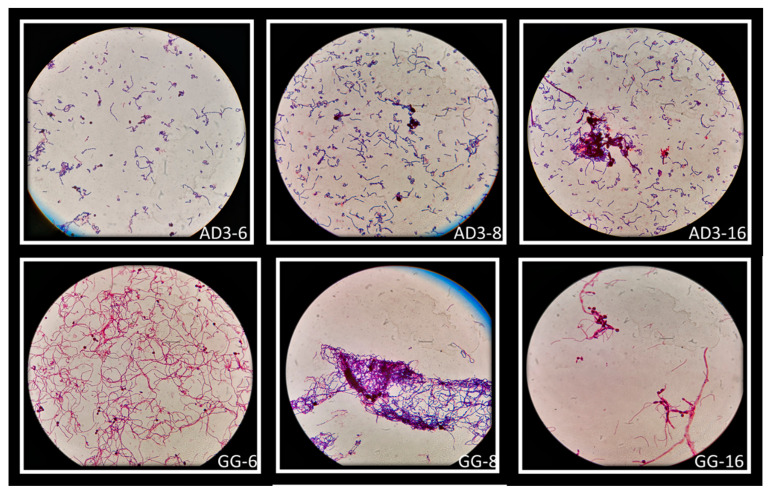
Macroscopically visible clumps after Giemsa staining of *L. rhamnosus* AD3 and GG with all three *Candida* strains. Co-aggregates of *L. rhamnosus* AD3 with *C. albicans* (AD3-6), *C. krusei* (AD3-8) and *C. tropicalis* (AD3-16). Co-aggregates of *L. rhamnosus* GG with *C. albicans* (GG-6), *C. krusei* (GG-8) and *C. tropicalis* (GG-16).

**Figure 8:**
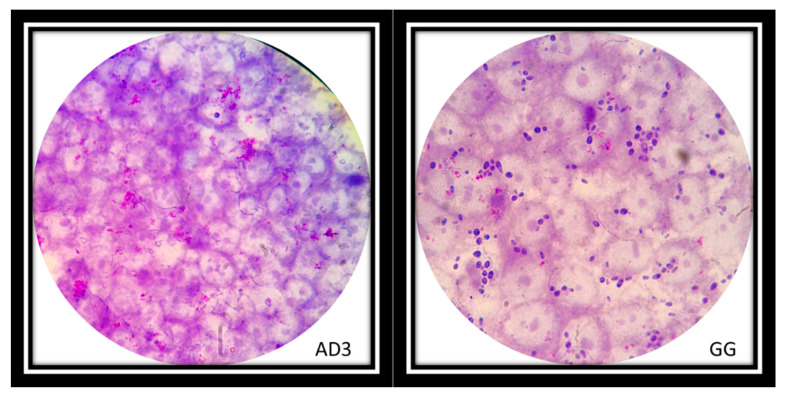
*L. rhamnosus* AD3 on Hep-2 cell monolayer inhibited adhesion of Candida on epithelial cells (AD3). Instead, the adhesion of *L. rhamnosus* GG on the Hep-2 monolayer did not inhibit the adhesion of the Candida (dark purple cells) (GG). Strains were observed under the microscope (1000×) after Giemsa staining.

The auto-aggregation ability of *Lactobacillus* strains was reflected in the ability to create co-aggregates with Candida cells. Vaginal strains were evaluated with *Candida albicans* (Figure 6A), *Candida krusei* (Figure 6B) and *Candida tropicalis* (Figure 6C). The co-aggregation scores were obtained after 4 h of incubation at 37 °C (Table 3). Figure 7 showed macroscopically visible clumps when *L. rhamnosus* AD3 and GG have been tested with all three *Candida* strains. It is possible that co-aggregates are able to create microenvironments around the pathogen capable of blocking its diffusion towards epithelial cells. Indeed, the study of the co-aggregation between *Lactobacillus* strains and *Candida* strains on Hep-2 and Caco-2 monolayers has shown, in some cases, how the bacteria-fungus co-aggregates inhibited the adhesion of *Candida* on epithelial cells (Figure 8 AD3). 

## 4. Discussion

Maintaining an adequate concentration of Lactobacilli is a necessary requirement to ensure the balance and health of the urogenital tract, especially in women [33]. 

Apparently, as already mentioned, alterations in the composition of the microbiota in women determine a greater susceptibility to infections. Therefore, re-establishing the Lactobacilli prevalence in the vaginal microbiota is necessary for the maintenance of the woman’s state of health [34].

The aim of this work was to find strains of Lactobacilli with probiotic characteristics adequate to respond to the physiological demands of women.

Firstly, the strains were tested for their susceptibility to antibiotics as well as for haemolytic and gelatinase activity. These tests were necessary to evaluate the safety of the strains, in fact no strain having antibiotic resistance genes or haemolytic/gelatinase activity can be marketed [22,32]. All the strains tested met these safety qualities.

The strains studied belonged to the following species: *L. crispatus*, *L. gasseri*, *L. rhamnosus* and *L. delbrueckii*. These species are among the most encountered in the vaginas of healthy women [35,36]. Usually, *L. crispatus* and *L. gasseri* are strong producers of lactic acid and inhibitory molecules against pathogenic species that cause urethritis, vaginitis or candidiasis [37,38]. In our study, a strain of *L. gasseri*, named SG3, showed the highest antagonistic activity against both bacteria and fungi, followed by *L. rhamnosus* AD3. Oddly enough, the positive control strain *L. rhamnosus* GG did not show any inhibitory activity. In addition, *L. gasseri* and *L. rhamnosus* are the species most resistant to gastrointestinal conditions, as well as strong producers of biofilm [23,24].

In this study, the strain that showed the greatest resistance at low pH and bile salts (pre- and post-exposition) was *L. rhamnosus* AD3, followed by *L. gasseri* SG3. In contrast, the positive control *L. rhamnosus* GG showed low resistance to these factors.

As far as biofilm production is concerned, all strains have been shown to be moderate or strong (AD3 and GG) producers except for CC1, BG2 and RB1. The latter did not even show resistance to gastrointestinal conditions proving to be the weakest strain among those tested and therefore the strain with less probiotic properties.

Moreover, the most of the strains under examination showed moderate accumulation of H_2_O_2_, with the exception of SG3 (very low) and BG2, EB3 and BA3 which showed to be great producers. However, the role of hydrogen peroxide as a protective agent is controversial. Indeed, despite the statement that *Lactobacillus* spp. can exert its probiotic properties also through the H_2_O_2_ production is accepted, there are several evidences as support the implausibility of “in vivo” action as an antimicrobial factor in the vaginal environment [39]. Hence, this property cannot be taken as a predisposing factor for a strain’s probiotic activity.

The ability of auto-aggregation of *Lactobacillus* strains can help to increase the colonization of vaginal environment by adhesion and sub-sequentially by biofilm formation and postbiotic production to inhibit the overgrowth pathogenic microorganisms [30,40]. Moreover, the ability to create co-aggregates with pathogenic microorganisms can modulate the destiny of pathologies among which stand out recurrent vulvovaginal candidiasis [41,42]. *L. rhamnosus* AD3 and *L. crispatus* CC4 showed the highest capacity to adhesion and auto-aggregation (Figure 5). Moreover, *L. rhamnosus* AD3 has shown an intended ability to form co-aggregates with all three Candida species, especially *C. tropicalis* (Figure 7). Although *L. rhamnosus* GG’s co-aggregation capacity is visibly greater (Figure 7), *L. rhamnosus* AD3 would seem capable of binding Candida cells more consistently. This property is evident from Candida absence on monolayer cells after co-culture exposure (Figure 8 AD3). On the contrary, bound Candida cells can be seen on cells despite the formation of co-aggregates with *L. rhamonsus* GG (Figure 8 GG). 

In summary, our results indicated that among all vaginal lactobacilli strains tested, *L. rhamnosus* AD3 revealed the required properties as potential probiotic strain. Indeed, it has responded to all safety features, and it has shown the highest levels of resistance to the gastrointestinal tract, production of active postbiotics and biofilm against fungi and bacteria, ability to adhere to epithelial mucosa, capacity to form strong aggregates and co-aggregates with Candida. Instead, *L. delbrueckii* RB1 proved to be the weakest strain. Strangely, as already said, the strain used as a positive control, *L. rhamonosus* GG, already on the market, showed low resistance to the gastrointestinal tract and no production of postbiotics active against pathogenic strains. Nonetheless, it showed high adhesion, auto-aggregation and co-aggregation ability, although candida cells were still present on the epithelial mucosa. 

These results suggest that *L. rhamnosus* AD3 may be a promising strain to aid vaginal microbiota recovery as complementary treatment option for urogenital diseases, and to enhance the normal vaginal microbiota in healthy young women.

Future studies are required to assess technological method to make pharmaceutical preparations able to respond to market demands. 

## Figures and Tables

**Table 1:** MIC values expressed as µg mL^−1^ for the evaluation of antibiotic resistance of the *Lactobacillus* strains.

Strains	AM	ERY	TET	GM	CM	CAF	S	KAN	CIP
BG 2 *L. crispatus*	≤0.03	0.12	0.25	1.0	0.12	0.12	2.0	2.0	>16
EB 1 *L. crispatus*	≤0.03	0.12	0.25	2.0	0.12	0.12	2.0	2.0	>16
EB 3 *L. crispatus*	≤0.03	0.25	0.50	2.0	0.12	0.12	2.0	1.0	>16
EB 4 *L. crispatus*	≤0.03	0.12	0.25	2.0	0.12	0.12	2.0	2.0	>16
CC 1 *L. crispatus*	≤0.03	0.12	0.25	4.0	0.12	0.25	4.0	2.0	>16
CC 4 *L. crispatus*	≤0.03	0.12	0.25	2.0	0.12	0.25	2.0	2.0	>16
BA 3 *L. gasseri*	0.12	0.06	1.0	2.0	0.12	0.12	2.0	2.0	>16
MS 1 *L. gasseri*	0.12	0.06	1.0	4.0	0.12	0.12	2.0	2.0	>16
SG 1 *L. gasseri*	0.12	0.06	1.0	4.0	0.12	0.12	2.0	2.0	>16
SG 3 *L. gasseri*	0.12	0.06	0.25	1.0	0.12	0.25	2.0	1.0	>16
RB 1 *L. delbrueckii*	0.5	0.06	0.25	2.0	0.25	0.25	4.0	2.0	>16
AD 3 *L. rhamnosus*	1.0	0.06	1.0	2.0	0.12	0.12	4.0	2.0	8
GG *L. rhamnosus*	1.0	0.06	1.0	4.0	0.12	0.25	4.0	2.0	8

AM ampicillin; ERY erythromycin; TET tetracycline; GM gentamicin; CM clindamycin; CAF chloramphenicol; S streptomycin; KAN kanamycin; CIP ciprofloxacin.

**Table 2:** Antimicrobial activity of cell-free supernatants produced by *Lactobacillus* strains against bacterial and fungal urogenital pathogens. Results were expressed in mm (mean ± SD).

Strains	*Escherichia coli*	*Enterococcus faecalis*	*Pseudomonas aeruginosa*	*Klebsiella pneumoniae*	*Streptococcus agalactiae*	*Candida albicans*	*Candida glabrata*	*Candida tropicalis*
BG 2 *L. crispatus*	≤6	18.3 ± 0.3	≤6	≤6	9.3 ± 0.3	10.2 ± 1.5	11.2 ± 1.7	17.7 ± 0.4
EB 1 *L. crispatus*	9.6 ± 1.9	23.5 ± 1.3	≤6	≤6	13.2 ± 1.6	16.3 ± 0.4	18.5 ± 1.5	22.1 ± 1.7
EB 3 *L. crispatus*	12.2 ± 0.2	28.5 ± 0.3	≤6	≤6	9.8 ± 1.6	22.3 ± 1.4	23.4 ± 1.8	19.6 ± 0.8
EB 4 *L. crispatus*	9.5 ± 1.3	16.7 ± 1.1	≤6	≤6	10.1 ± 2.2	20.5 ± 2.3	24.5 ± 0.7	21.2 ± 1.6
CC 1 *L. crispatus*	9.7 ± 0.3	17.4 ± 0.2	12.4 ± 0.2	9.5 ± 0.5	11.2 ± 0.5	18.4 ± 0.4	21.4 ± 0.3	19.1 ± 0.6
CC 4 *L. crispatus*	10.0 ± 2.3	24.3 ± 0.3	23.6 ± 0.1	18.5 ± 0.6	11.5 ± 0.4	19.6 ± 0.9	21.3 ± 2.4	20.5 ± 0.5
BA 3 *L. gasseri*	12.3 ± 0.4	20.3 ± 0.4	23.5 ± 0.2	14.4 ± 0.2	12.3 ± 1.1	17.9 ± 1.4	15.5 ± 2.4	20.1 ± 1.3
MS 1 *L. gasseri*	≤6	19.3 ± 1.3	21.4 ± 1.7	20.4 ± 0.8	12.5 ± 1.9	20.3 ± 1.7	21.6 ± 2.3	21.5 ± 3.6
SG 1 *L. gasseri*	≤6	20.4 ± 0.3	15.5 ± 0.6	≤6	11.3 ± 0.4	20.2 ± 0.4	21.6 ± 0.5	21.3 ± 0.3
SG 3 *L. gasseri*	10.1 ± 0.2	22.3 ± 0.4	26.4 ± 0.3	25.3 ± 0.2	17.6 ± 0.3	21.4 ± 0.6	22.4 ± 0.3	25.4 ± 0.1
RB 1 *L. delbrueckii*	≤6	≤6	≤6	≤6	≤6	≤6	≤6	≤6
AD 3 *L. rhamnosus*	≤6	21.4 ± 0.5	20.4 ± 1.4	16.4 ± 0.3	11.5 ± 2.3	17.4 ± 1.2	21.3 ± 0.5	≤6
GG *L. rhamnosus*	≤6	≤6	≤6	≤6	≤6	≤6	≤6	≤6

**Table 3:** Adhesion of *Lactobacillus* strains to Hep-2 cells and Caco-2 [29] and co-aggregation rates obtained with three different strains of *Candida* [31].

Strains	Adhesion on Hep-2 (n Bacteria/Cell)	Adhesion on Caco-2 (n Bacteria/Cell)	*Candida albicans* *	*Candida krusei* *	*Candida tropicalis* *
BG 2 *L. crispatus*	37 ± 12	43 ± 11	1	1	2
EB 1 *L. crispatus*	50 ± 15	50 ± 14	1	1	3
EB 3 *L. crispatus*	33 ± 13	38 ± 4	1	2	2
EB 4 *L. crispatus*	39 ± 8	41 ± 8	2	2	2
CC 1 *L. crispatus*	31 ± 10	35 ± 11	1	2	3
CC 4 *L. crispatus*	49 ± 6	49 ± 9	2	2	3
BA 3 *L. gasseri*	24 ± 5	31 ± 10	2	2	3
MS 1 *L. gasseri*	24 ± 13	37 ± 11	1	1	4
SG 1 *L. gasseri*	25 ± 9	29 ± 15	2	1	2
SG 3 *L. gasseri*	30 ± 11	43 ± 12	1	1	2
RB1 *L. delbrueckii*	30 ± 3	45 ± 16	2	1	3
AD3 *L. rhamnosus*	82 ± 12	108 ± 20	1	2	4
GG *L. rhamnosus*	45 ± 15	50 ± 16	1	4	2

* co-aggregation rates: No aggregation (0); small aggregates (1); aggregates with larger numbers of bacteria (2); macroscopically visible clumps with larger groups of bacteria (3) and macroscopically visible clumps (4).

## Data Availability

Data is contained within the article.

## References

[1] Krepelka P., Fait T., Urbankova I., Hanacek J., Krofta L., Dvorak V. (2020). Risky sexual behaviour and contraceptive use among young women in the czech republic. Cent. Eur. J. Public Health.

[2] Lanis A., Talib H.J., Dodson N. (2020). Prepubertal and adolescent vulvovaginitis: What to do when a girl reports vaginal discharge. Pediatr. Ann..

[3] Brotman R.M., Erbelding E.J., Jamshidi R.M., Klebanoff M.A., Zenilman J.M., Ghanem K.G. (2007). Findings associated with recurrence of bacterial vaginosis among adolescents attending sexually transmitted diseases clinics. J. Pediatr. Adolesc. Gynecol..

[4] Zeng X.L., Zhang Y.F., Zhang T.H., Xue Y., Xu H.Q., An R.F. (2018). Risk factors of vulvovaginal candidiasis among women of reproductive age in xi’an: A cross-sectional study. BioMed Res. Int..

[5] Marelli G., Papaleo E., Ferrari A. (2004). Lactobacilli for prevention of urogenital infections: A review. Eur. Rev. Med. Pharmacol. Sci..

[6] Zowawi H.M., Harris P.N., Roberts M.J., Tambyah P.A., Schembri M.A., Pezzani M.D., Williamson D.A., Paterson D.L. (2015). The emerging threat of multidrug-resistant gram-negative bacteria in urology. Nat. Rev. Urol..

[7] Sobel J.D., Sobel R. (2018). Current treatment options for vulvovaginal candidiasis caused by azole-resistant candida species. Expert Opin. Pharmacother..

[8] Lewis A.L., Gilbert N.M. (2020). Roles of the vagina and the vaginal microbiota in urinary tract infection: Evidence from clinical correlations and experimental models. GMS Infect. Dis..

[9] Barrientos-Duran A., Fuentes-Lopez A., de Salazar A., Plaza-Diaz J., Garcia F. (2020). Reviewing the composition of vaginal microbiota: Inclusion of nutrition and probiotic factors in the maintenance of eubiosis. Nutrients.

[10] Gajer P., Brotman R.M., Bai G., Sakamoto J., Schutte U.M., Zhong X., Koenig S.S., Fu L., Ma Z.S., Zhou X. (2012). Temporal dynamics of the human vaginal microbiota. Sci. Transl. Med..

[11] Fuochi V., Li Volti G., Furneri P.M. (2017). Commentary: Lactobacilli dominance and vaginal ph: Why is the human vaginal microbiome unique?. Front. Microbiol..

[12] Petricevic L., Domig K.J., Nierscher F.J., Sandhofer M.J., Krondorfer I., Kneifel W., Kiss H. (2013). Differences in the vaginal lactobacilli of postmenopausal women and influence of rectal lactobacilli. Climacteric.

[13] Vodstrcil L.A., Twin J., Garland S.M., Fairley C.K., Hocking J.S., Law M.G., Plummer E.L., Fethers K.A., Chow E.P., Tabrizi S.N. (2017). The influence of sexual activity on the vaginal microbiota and gardnerella vaginalis clade diversity in young women. PLoS ONE.

[14] Xie H.Y., Feng D., Wei D.M., Mei L., Chen H., Wang X., Fang F. (2017). Probiotics for vulvovaginal candidiasis in non-pregnant women. Cochrane Database Syst. Rev..

[15] Donders G., Bellen G., Oerlemans E., Claes I., Ruban K., Henkens T., Kiekens F., Lebeer S. (2020). The use of 3 selected lactobacillary strains in vaginal probiotic gel for the treatment of acute candida vaginitis: A proof-of-concept study. Eur. J. Clin. Microbiol. Infect. Dis..

[16] Qian Z., Zhao D., Yin Y., Zhu H., Chen D. (2020). Antibacterial activity of lactobacillus strains isolated from mongolian yogurt against gardnerella vaginalis. BioMed Res. Int..

[17] Borchert D., Sheridan L., Papatsoris A., Faruquz Z., Barua J.M., Junaid I., Pati Y., Chinegwundoh F., Buchholz N. (2008). Prevention and treatment of urinary tract infection with probiotics: Review and research perspective. Indian J. Urol..

[18] Bruce A.W., Reid G. (1988). Intravaginal instillation of lactobacilli for prevention of recurrent urinary tract infections. Can. J. Microbiol..

[19] Reid G., Millsap K., Bruce A.W. (1994). Implantation of lactobacillus-casei var rhamnosus into vagina. Lancet.

[20] MinisterodellaSalute (2013). Linee Guida su Probiotici e Prebiotici.

[21] Fuochi V., Li Volti G., Furneri P.M. (2017). Probiotic properties of lactobacillus fermentum strains isolated from human oral samples and description of their antibacterial activity. Curr. Pharm. Biotechnol..

[22] EFSA (2012). Panel on additives and products or substances used in animal feed (feedap). Guidance on the assessment of bacterial susceptibility to antimicrobials of human and veterinary importance. EFSA J..

[23] Fuochi V., Cardile V., Petronio Petronio G., Furneri P.M. (2019). Biological properties and production of bacteriocins-like-inhibitory substances by lactobacillus sp. Strains from human vagina. J. Appl. Microbiol..

[24] Fuochi V., Petronio G.P., Lissandrello E., Furneri P.M. (2015). Evaluation of resistance to low ph and bile salts of human lactobacillus spp. Isolates. Int. J. Immunopathol. Pharmacol..

[25] CLSI (2019). M100 s29 Performance Standards for Antimicrobial Susceptibility Testing.

[26] CLSI (2017). M60 Performance Standards for Antifungal Susceptibility Testing of Yeasts.

[27] Magaldi S., Mata-Essayag S., Hartung de Capriles C., Perez C., Colella M.T., Olaizola C., Ontiveros Y. (2004). Well diffusion for antifungal susceptibility testing. Int. J. Infect. Dis..

[28] Fuochi V., Coniglio M.A., Laghi L., Rescifina A., Caruso M., Stivala A., Furneri P.M. (2019). Metabolic characterization of supernatants produced by lactobacillus spp. With in vitro anti-legionella activity. Front. Microbiol..

[29] Furneri P.M., Garozzo A., Musumarra M.P., Scuderi A.C., Russo A., Bonfiglio G. (2003). Effects on adhesiveness and hydrophobicity of sub-inhibitory concentrations of netilmicin. Int. J. Antimicrob. Agents.

[30] Gil N.F., Martinez R.C., Gomes B.C., Nomizo A., De Martinis E.C. (2010). Vaginal lactobacilli as potential probiotics against candida spp.. Braz. J. Microbiol..

[31] Reid G., McGroarty J.A., Domingue P.A.G., Chow A.W., Bruce A.W., Eisen A., Costerton J.W. (1990). Coaggregation of urogenital bacteria in vitro and in vivo. Curr. Microbiol..

[32] FAO (2006). Probiotic in Food: Health and Nutrition Properties and Guidelines for Evaluation.

[33] Marconi C., El-Zein M., Ravel J., Ma B., Lima M.D., Carvalho N.S., Alves R.R.F., Parada C., Leite S.H.M., Giraldo P.C. (2020). Characterization of the vaginal microbiome in women of reproductive age from five regions in brazil. Sex. Transm. Dis..

[34] Collins S.L., McMillan A., Seney S., van der Veer C., Kort R., Sumarah M.W., Reid G. (2018). Promising prebiotic candidate established by evaluation of lactitol, lactulose, raffinose, and oligofructose for maintenance of a lactobacillus-dominated vaginal microbiota. Appl. Environ. Microbiol..

[35] Alioua S., Abdi A., Fhoula I., Bringel F., Boudabous A., Ouzari I.H. (2016). Diversity of vaginal lactic acid bacterial microbiota in 15 algerian pregnant women with and without bacterial vaginosis by using culture independent method. J. Clin. Diagn. Res..

[36] Mehta O., Ghosh T.S., Kothidar A., Gowtham M.R., Mitra R., Kshetrapal P., Wadhwa N., Thiruvengadam R., Nair G.B., GARBH-Ini study group (2020). Vaginal microbiome of pregnant indian women: Insights into the genome of dominant lactobacillus species. Microb. Ecol..

[37] Abdelmaksoud A.A., Koparde V.N., Sheth N.U., Serrano M.G., Glascock A.L., Fettweis J.M., Strauss J.F., Buck G.A., Jefferson K.K. (2016). Comparison of lactobacillus crispatus isolates from lactobacillus-dominated vaginal microbiomes with isolates from microbiomes containing bacterial vaginosis-associated bacteria. Microbiology.

[38] Tortelli B.A., Lewis W.G., Allsworth J.E., Member-Meneh N., Foster L.R., Reno H.E., Peipert J.F., Fay J.C., Lewis A.L. (2020). Associations between the vaginal microbiome and candida colonization in women of reproductive age. Am. J. Obstet. Gynecol..

[39] Tachedjian G., O’Hanlon D.E., Ravel J. (2018). The implausible “in vivo” role of hydrogen peroxide as an antimicrobial factor produced by vaginal microbiota. Microbiome.

[40] Klopper K.B., Deane S.M., Dicks L.M.T. (2018). Aciduric strains of lactobacillus reuteri and lactobacillus rhamnosus, isolated from human feces, have strong adhesion and aggregation properties. Probiotics Antimicrob. Proteins.

[41] Rastogi S., Mittal V., Singh A. (2019). In vitro evaluation of probiotic potential and safety assessment of lactobacillus mucosae strains isolated from donkey’s lactation. Probiotics Antimicrob. Proteins.

[42] Jang S.J., Lee K., Kwon B., You H.J., Ko G. (2019). Vaginal lactobacilli inhibit growth and hyphae formation of candida albicans. Sci. Rep..

